# *FGFR2* Point Mutations in 466 Endometrioid Endometrial Tumors: Relationship with MSI, *KRAS*, *PIK3CA*, *CTNNB1* Mutations and Clinicopathological Features

**DOI:** 10.1371/journal.pone.0030801

**Published:** 2012-02-23

**Authors:** Sara A. Byron, Michael Gartside, Matthew A. Powell, Candice L. Wellens, Feng Gao, David G. Mutch, Paul J. Goodfellow, Pamela M. Pollock

**Affiliations:** 1 Cancer and Cell Biology Division, Translational Genomics Research Institute, Phoenix, Arizona, United States of America; 2 Divisions of Gynecologic Oncology, Biostatistics and Endocrine Oncology, Siteman Cancer Center and Washington University School of Medicine, St. Louis, Missouri, United States of America; National Cancer Center, Japan

## Abstract

Mutations in multiple oncogenes including *KRAS*, *CTNNB1*, *PIK3CA* and *FGFR2* have been identified in endometrial cancer. The aim of this study was to provide insight into the clinicopathological features associated with patterns of mutation in these genes, a necessary step in planning targeted therapies for endometrial cancer. 466 endometrioid endometrial tumors were tested for mutations in *FGFR2*, *KRAS*, *CTNNB1*, and *PIK3CA*. The relationships between mutation status, tumor microsatellite instability (MSI) and clinicopathological features including overall survival (OS) and disease-free survival (DFS) were evaluated using Kaplan-Meier survival analysis and Cox proportional hazard models. Mutations were identified in *FGFR2* (48/466); *KRAS* (87/464); *CTNNB1* (88/454) and *PIK3CA* (104/464). *KRAS* and *FGFR2* mutations were significantly more common, and CTNNB1 mutations less common, in MSI positive tumors. *KRAS* and *FGFR2* occurred in a near mutually exclusive pattern (p = 0.05) and, surprisingly, mutations in *KRAS* and *CTNNB1* also occurred in a near mutually exclusive pattern (p = 0.0002). Multivariate analysis revealed that mutation in *KRAS* and *FGFR2* showed a trend (p = 0.06) towards longer and shorter DFS, respectively. In the 386 patients with early stage disease (stage I and II), *FGFR2* mutation was significantly associated with shorter DFS (HR = 3.24; 95% confidence interval, CI, 1.35–7.77; p = 0.008) and OS (HR = 2.00; 95% CI 1.09–3.65; p = 0.025) and *KRAS* was associated with longer DFS (HR = 0.23; 95% CI 0.05–0.97; p = 0.045). In conclusion, although *KRAS* and *FGFR2* mutations share similar activation of the MAPK pathway, our data suggest very different roles in tumor biology. This has implications for the implementation of anti-FGFR or anti-MEK biologic therapies.

## Introduction

Endometrial cancer comprises about 4% of cancer in women globally, with higher incidence in developed countries. The American Cancer Society estimates endometrial cancer will be the fourth most common cancer diagnosed and the eighth leading cause of cancer deaths in women in 2010 [1]. Approximately 80% of women are diagnosed with early stage cancers, clinically confined to the uterus. Early diagnosis of endometrial cancer contributes to the relatively good overall long-term survival. However, for women who present with late stage disease or who suffer recurrences, outcomes are poor. The five-year survival for women with recurrent, progressive or metastatic endometrial cancer is estimated as only 13% [2].

Considerable effort has gone into developing systems to more effectively identify patients with endometrioid endometrial cancer that carry an elevated risk of recurrence so they can be targeted for adjuvant therapies (radiation, hormonal therapy, chemotherapy or combination therapies). Those patients that present with extrauterine disease (stage III/IV) carry a high risk of recurrence and progression. The majority of patients (∼80%), however, present with tumors clinically confined to the uterus (stage I/II). In these early stage patients, multiple studies have shown that the risk of recurrence is associated with tumor grade, depth of myometrial invasion, occult extension into the cervix and tumor cell invasion of lymphatic vessels (lymphovascular space invasion: LVSI), where high grade is the most widely accepted adverse prognostic marker [2], [3]. The identification of molecular prognostic markers that could be incorporated into a risk stratification model is an unmet clinical need.

Since 1988, the International Federation of Gynecology and Obstetrics (FIGO) has recommended full systematic pelvic and para-aortic lymphadectomy as part of staging for endometrial cancer. A new 2009 FIGO staging system has recently been implemented where tumors with no evidence of myometrial invasion are combined with tumors that show invasion to less than 50% of the myometrium and grouped into stage 1A [4]. There is considerable controversy in the literature as to the benefit of lymphadectomy (measured as disease-free and overall survival) in management of endometrial cancer patients. Some of the conflicting results may reflect difference in study designs and analysis methods. Some studies have reported improved survival in those patients with early stage cancers but only in those with high histologic grade [5]. More recently, there have been several large multicenter clinical trials that have indicated systematic pelvic lymphadectomy does not improve disease free or overall survival [6], [7]. Thus, for many patients in the United States and most patients worldwide, lymph nodes are not removed and patients are treated based on uterine risk factors alone. The development of prognostic markers that could be used for risk stratification and to inform subsequent treatment options is clearly needed for early stage patients.

FGFR2 has been shown to be activated in a number of cancers due to gene amplification [8], [9], [10] and point mutation [11], [12], [13]. Our group previously reported somatic activating *fibroblast growth factor receptor 2* (*FGFR2*) mutations in 18/115 (16%) endometrioid endometrial cancers [14]. Two independent studies subsequently reported a mutation frequency of 10% [11], [15]. In our initial analysis of 115 cases there was over-representation of higher stage cancers that subsequently recurred and of tumors that had lost DNA mismatch repair (MSI-positive cancers). The objective of the current study was to determine the prevalence of *FGFR2, CTNNB1, KRAS and PIK3CA* mutation in a large, unselected cohort of endometrioid endometrial cancers and to determine the relationship between mutation status and clinicopathologic variables including outcome. Mutations in PTEN were not included in this analysis due to the increased cost associated with sequencing all 9 exons of this tumor suppressor gene. In addition, the high prevalence of PTEN aberration (70%) argued against a possible association with poor prognosis in this tumor type.

## Materials and Methods

### Ethics statement

All research subjects provided written consent to ongoing protocols 91-507 and 93-0828, approved by the Washington University's Human Research Protection Office continuing Review Committee. The work performed at TGen was determined to be exempt from IRB approval following review and receipt of a Verification of Protections for Human research subjects form signed by Dr Goodfellow and a copy of the blank consent form.

### Study participants and clinical data

Tumor specimens were prospectively collected at the time of hysterectomy (1991–2006) for patients treated by the Division of Gynecologic Oncology at Washington University School of Medicine/Barnes–Jewish Hospital. Surgical staging and tumor grade was assigned on the basis of FIGO 1988. Patients who had received preoperative radiation or chemotherapy were excluded from analysis. The prospectively collected clinical and pathologic information was stored in a computerized database. Following their initial treatment, these patients were routinely followed at 3-month intervals for the first 2 years and then at 6-month intervals for at least 3 years. Disease surveillance included physical examination and periodic pap smears. Diagnostic imaging and directed biopsies were performed as clinically indicated. Histological confirmation of all recurrences was performed. Follow-up data were abstracted from clinic charts, hospital records, and the Siteman Cancer Center/Barnes-Jewish Hospital's cancer registry.

Patients for whom follow-up data were unavailable or who died perioperatively (within 30 days of hysterectomy) were excluded from the analyses. The study population comprised 466 patients with endometrioid endometrial cancer, 386 of which had disease confined to the uterus (stage I or II).

### Tissue processing, FGFR2 mutation analysis

Tissue specimens and blood were obtained at the time of surgery, snap frozen, and stored at −70°C. Tumors were evaluated to select tissues with >66% neoplastic cellularity for DNA preparations. DNA was isolated using proteinase K and phenol extraction or the DNeasy Tissue Kit (Qiagen Inc, Valencia, CA). DNA was extracted from peripheral blood leukocytes or, when blood was not available, from uninvolved myometrium, as previously described [16], [17].

Exons 7, 8, 10, 13 and 15 of *FGFR2*, exon 2 of *KRAS*, exon 3 of CTNNB1, and exons 9 and 20 of *PIK3CA* were tested for mutations by direct sequencing. PCR primers and conditions are available upon request [18], [19]. Sequences were analyzed using Sequencher (Gene Codes, Ann Arbor, MI). Mutation analysis was performed on blinded samples. All potential mutations were confirmed with repeat amplification and sequencing of the exon of interest. Matched normal DNA was analyzed to confirm the mutation arose somatically for all mutations in *FGFR2* and *KRAS* and *CTNNB1*. For *PIK3CA*, rare and novel mutations were confirmed to have arisen somatically and common tumor-associated mutations were confirmed in the majority of samples.

### Microsatellite instability (MSI) testing

MSI analysis is routinely performed for all tumors. The MSI status and methods used for the majority of the cases reported here have been previously described [20].

### Statistical analysis

The relationship between gene mutation status and covariates was assessed using Fisher's exact test or Student's t-test as appropriate. Overall survival (OS) was defined as the time from date of surgery to death due to any cause. Survivors were censored at the date of last contact. Disease free survival (DFS) was defined as the time from surgery to recurrence or progression. Patients were excluded if they had died within 30 days of surgery. The Kaplan-Meier product limit method was used to estimate OS and DFS. Univariate and multivariate Cox proportional hazard models were fitted to assess the effects of the covariates on OS and DFS, and the proportional hazard assumptions were checked using scaled Schoenfeld residuals [21]. Clinically accepted poor prognostic covariates that were significant on univariate analysis were included in the model including stage, grade and age. In the analysis of DFS, Gray's competing risk methods were also used to account for the potential competing effect of death [22]. All analyses were two-sided and significance was set at a *p*-value of 0.05. Statistical analyses were performed using SAS (SAS Institutes, Cary, NC), as well as the cmprsk R (http://biowww.dfci.harvard.edu/~gray) statistical packages for competing risk analysis.

## Results

The mean age at diagnosis for the 466 cases analyzed was 63.7 years with a mean follow-up time of 70.2 months (0.7–176). The majority of patients presented with early-stage disease (386 or 83% stage I or II) (Table 1). Mutation analysis was successful for the four genes of interest as follows: *FGFR2* (466 tumors, 100%); *KRAS* and *PIK3CA* (464 tumors, 99%); and *CTNNB1* (454 tumors, 97%). Mutation data for all four genes was obtained for 453 cases (97%).

**Table 1:** Patient Demographics and Clinicopathologic Characteristics.

Clinicopathologic Category	Subcategory	Entire Cohort of 466 Endometrioid Endometrial Tumors	Cohort of 386 Low Stage Endometrioid Endometrial Tumors
Mean Age at Diagnosis (SD)		63.7 (11.7)	63.5 (11.6)
Follow-up Time (Mean)		70.2 months (0.7–176)	75.4 months (1.4–176)
Race	Caucasian/Asian	411 (88%)	338 (88%)
	African American	55 (12%)	48 (12%)
FIGO Stage	1A	85 (18%)	85 (22%)
	1B	192 (41%)	192 (50%)
	1C	71 (15%)	71 (18%)
	IIA	18 (4%)	18 (5%)
	IIB	20 (4%)	20 (5%)
	III	62 (13%)	-
	IV	18 (4%)	-
Grade	1	249 (53%)	225 (58%)
	2	152 (33%)	122 (32%)
	3	65 (14%)	39 (10%)
Recurrence	No	399 (86%)	353 (91%)
	Yes	67 (14%)	33 (8.5%)
Vital Status	Alive	318 (68%)	283 (73%)
	Dead	148 (32%)	103 (27%)
MSI	No	308 (66%)	257 (67%)
	Yes	158 (34%)	129 (33%)
*FGFR2* Mutation	No	418 (90%)	347 (90%)
	Yes	48 (10%)	39 (10%)
*KRAS* Mutation	No	377 (81%)	311 (81%)
	Yes	87 (19%)	73 (19%)
*CTNNB1* Mutation	No	366 (81%)	298 (79%)
	Yes	88 (19%)	78 (21%)
*PIK3CA* Mutation	No	360 (78%)	291 (76%)
	Yes	104 (22)	93 (24%)

### Prevalence and spectrum of FGFR2 mutations

**Figure 1:**
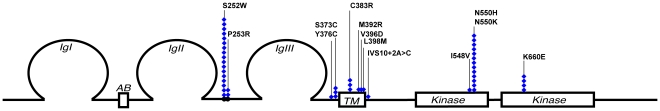
Schematic figure of FGFR2 mutations identified in endometrioid endometrial tumors. Blue diamonds indicate each instance of a mutation in the Washington University School of Medicine cohort. Mutations are numbered relative to *FGFR2*b (NP_075259.2). Mutations at 6 codons (S252, P253, Y376, C383, N550, K660) comprise >90% of all mutations identified.

We identified *FGFR2* mutations in 48/466 (10.3%) tumors (Table S1), including 115 previously investigated cases [18]. One *FGFR2* sequence alteration we originally reported as a frameshift (c.2287-88delCT) was excluded from analyses because of uncertainty as to whether the sequence change was functionally significant. The most common mutations were S252W (n = 18; 37%) and N550K (n = 12, 25%). All together, 7 mutations affecting 6 codons (S252W, P253R, Y376C, C383R, N550K, N550H and K660E) accounted for 90% of the mutations identified (Figure 1). We identified two additional novel mutations in the transmembrane domain not previously described (V396D and L398M), both of which we presume to be pathogenic. The valine at FGFR2 codon 396 is highly conserved across species and between FGFR1-FGFR3 family members. Furthermore, similar substitutions in the transmembrane region of FGFR3 have been shown to be activating. Replacement of a hydrophobic residue with a glutamic acid in FGFR3 (A391E) has been identified both in the germline of patients with Crouzon syndrome [23] and as a somatic mutation in bladder cancer [24]. Functional studies have indicated the A391E mutation stabilizes the active dimer via hydrogen bonds [25]. We also hypothesize that by analogy the L398M mutation (a conservative substitution resulting in the introduction of a larger hydrophobic residue) is similarly pathogenic. This mutation may result in a structural change leading to a more active conformation, or may promote receptor activation independent of structural changes e.g. altered protein turnover as has been shown for the G380R mutation in FGFR3 [26]. Functional studies will be required to conclusively confirm these mutations result in receptor activation.

### Prevalence and spectrum of KRAS mutations

We identified mutations at codons 12 and 13 in *KRAS* in 87/464 (19%) samples, including 115 previously investigated cases [19]. The two most common mutations were G12D (33%) and G12V (29%), which is similar to the frequencies observed in the Catalog of Somatic Mutations in Cancer (COSMIC) (39% and 22%, respectively) in endometrial tumors. All mutations observed had been reported previously (Table S2).

### Prevalence and spectrum of *PIK3CA* mutations

We identified 29 different mutations in exon 9 and 20 of *PIK3CA* in a total of 104/464 (22%) cases (Table S3). The majority of these (65/104, 63%) occurred in the kinase domain encoded by exon 20 with the two most common mutations being E545K and H1047R. We identified 2 novel mutations in exon 20, L1006F and Q1014H. These non-conservative missense changes occurred in the highly conserved C-terminal portion of the protein. *In silico* predictions using SIFT indicate L1006F would be tolerated but Q1014H would not, whereas PolyPhen classifies L1006F as possibly damaging and Q1014H as benign. Although, in the absence of functional studies, the caveat exists that these mutations may indeed be passenger mutations and impart no increased “fitness” to the tumor, they were included in the current statistical analysis as pathogenic given that the functional validation of many more common mutations as oncogenic has not been reported.

### Prevalence and spectrum of *CTNNB1* mutations

We identified 21 different mutations in *CTNNB1* in 88/454 (19%) endometrioid tumors (Table S4). The three most common mutations occurred at D32Y (13%), S33C (11%), S37F (17%). All mutations had been reported previously.

### Prevalence of microsatellite instability and association with mutations

**Figure 2:**
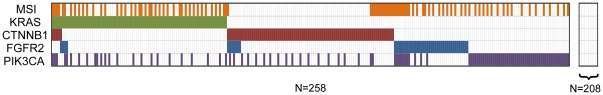
Pattern of *KRAS, CTNNB1, FGFR2*, *PIK3CA* mutations and MSI status in 466 endometrioid endometrial tumors. Gene mutations and MSI positive status are depicted by colored bars. 258 tumors had a mutation in at least one of the genes evaluated, whereas 208 tumors did not demonstrate mutation of *KRAS, CTNNB1, FGFR2, or PIK3CA*.

158/466 (34%) of tumors were MSI positive. Mutations in *KRAS* were significantly more common in MSI positive tumors (42/158; 28%) compared to microsatellite stable (MSS) tumors (45/306; 14%) (p = 0.003, Fisher's exact test). Similarly, mutations in *FGFR2*, were significantly more common in MSI positive tumors (24/158; 15%) compared to MSS tumors (24/308; 8%) (p = 0.016). In contrast, mutations in *CTNNB1* were significantly less common in MSI positive tumors (17/152; 11%) compared to MSS tumors (71/302; 24% p = 0.002). Mutations in *PIK3CA* were more common in MSI positive tumors (43/158; 27%) compared to MSS tumors (61/306; 20%), although this was not significant (p = 0.08). Figure 2 summarizes the patterns of mutations and association with MSI status.

Based on our understanding of receptor tyrosine kinase-MAPK signaling, and our preliminary analysis of 115 endometrial tumors, we anticipated that *FGFR2* and *KRAS* mutations would occur in a mutually exclusive pattern. Indeed, only 4/87 (5%) *KRAS* mutation-positive tumors carried a *FGFR2* mutation (S252W x2, P253R, L398M), whereas 44/377 (12%) *KRAS* mutation negative tumors carried an *FGFR2* mutation (p = 0.05, two-tailed Fisher's exact test). To investigate whether the tumors carrying mutations in both *FGFR2* and *KRAS* were polyclonal, DNA from a different portion of the tumor was extracted from archived paraffin tissue and in all four cases both mutations were confirmed.

Perhaps the most surprising finding from this cohort is that mutations in *KRAS* and *CTNNB1* demonstrated a similar pattern of mutual exclusivity and rarely occurred together. In the 453 tumors sequenced for both genes, 88 and 85 carried mutations in *CTNNB1* and *KRAS*, respectively. Of those tumors with *CTNNB1* mutations, only 5/88 (5.7%) carried *KRAS* mutations, whereas 80/365 (22%) of the *CTNNB1*-wildtype tumors carried a *KRAS* mutation (p = 0.0002, two-tailed Fisher's exact test). Given *CTNNB1* mutations were significantly more common in MSS tumors, we looked for the relationship between *KRAS* and *CTNNB1* mutations in both MSS and MSI tumors. This association was even stronger in those tumors that demonstrated microsatellite stability where 1/71 (1%) *CTNNB1* mutation positive tumors carried a *KRAS* mutation, whereas 44/230 (19%) of the *CTNNB1* wildtype tumors carried a *KRAS* mutation (p = 0.00004, two-tailed Fisher's exact test). In contrast, this association was not present in those tumors with MSI as 4/17 (24%) *CTNNB1* mutation positive tumors carried an activating *KRAS* mutation whereas 36/135 (27%) of the *CTNNB1* wildtype tumors carried a *KRAS* mutation.

Surprisingly, given the near mutual exclusivity of *FGFR2* and *KRAS*, and of *CTNNB1* and *KRAS*, no such pattern was seen for *FGFR2* and *CTNNB1*. Specifically 8/88 (9%) *CTNNB1* mutation positive tumors carried an *FGFR2* mutation, whereas 40/365 (11%) *CTNNB1* wildtype tumors carried an *FGFR2* mutation. Within the MSS cohort of tumors, 7/71 (10%) *CTNNB1* mutation positive tumors carried an *FGFR2* mutation whereas 17/230 (7%) of the *CTNNB1* wildtype tumors carried an *FGFR2* mutation.

### Association of mutations with clinicopathologic features

There was no association between *FGFR2*, *KRAS*, *PIK3CA* mutation and age at diagnosis. *CTNNB1* mutations were, however, significantly more common in patients diagnosed before age 60 (49/183, 27%) compared to those diagnosed after age 60 (39/271, 14%) (p = 0.0016, two-tailed Fisher's exact test). We chose 60 as our age cutoff based on previous data indicating reduced survival in patients >60 [2]. There was no association between mutations in any of the four oncogenes investigated and patient race. *FGFR2* mutations were more common in Caucasian/Asian cases (46/411, 11%) than African American patients (2/55, 3%), albeit this was not significant (p = 0.10). *PIK3CA* mutations were significantly more common in stage I/II tumors (93/384, 24%) compared to late stage tumors (11/80, 13%) (p = 0.04, two tailed Fisher's exact test) (Table S5). *CTNNB1* mutations were significantly associated with low tumor grade: grade 1, 59/243, (24%); grade 2, 25/149 (17%); grade 3, 4/62 (6%) (p = 0.0027, two-tailed Fisher's exact test) and *FGFR2* mutations showed a trend towards an association with grade (grade 1, 29/249 (12%); grade 2 17/152 (11%); grade 3, 2/65 (3%) (p = 0.10) (Table S6). As well and moderately differentiated (grade 1,2) tumors have been shown to share a similar genetic etiology, we also compared mutation frequency in this group compared to high grade tumors. When analyzed in this way, *CTNNB1* mutations were significantly less common in high grade tumors, 4/62 (6%) compared to lower grade tumors 84/392, (21%) (p = 0.004, two-tailed Fisher's exact test) as were *FGFR2* mutations (grade 1/2, 46/401 (11%); grade 3, 2/65 (3%) (p = 0.04, two-tailed Fisher's exact test).

### Mutations, patient outcome and other clinicopathologic features

Mutation status for the four oncogenes investigated was not associated with overall survival (OS) in the total cohort of 466 cases. OS was associated with age >60 (p = 0.0002), advanced stage (III/IV) (p<0.0001), FIGO tumor grade 2 (p = 0.0014), FIGO grade 3, p<0.0001) and adjuvant therapy (p<0.0001) (Table 2). Multivariate analysis did not indicate that the mutation status of any gene was associated with OS but age >60 yrs, advance stage and higher grade remained significantly associated with shorter OS (Table 2, data not shown).

**Table 2:** Hazard Ratio (HR) and 95% Confidence Interval (CI) for Cohort of 466 Endometrioid Endometrial Cancers.

Univariate Analyses						
	Disease Free Survival			Overall Survival		
	HR Ratio	95% CI	P	HR Ratio	95% CI	P
Age >60	1.47	0.88–2.45	0.14	2.01	1.39–2.92	0.0002
Race (Black)	1.36	0.70–2.66	0.37	1.39	0.88–2.19	0.16
FIGO stage IA/1B	REF			REF		
FIGO stage IC	2.61	1.18–5.74	0.018	1.403	0.87–2.27	0.17
FIGO stage II	3.26	1.34–7.93	0.009	2.10	1.21–3.64	0.0083
FIGO stage III/IV	6.80	4.20–11.0	<0.0001	3.79	2.65–5.42	<0.0001
FIGO Grade 2	2.71	1.45–5.07	0.0019	1.85	1.27–2.70	0.0014
FIGO Grade 3	7.91	4.24–14.77	<0.0001	4.34	2.85–6.60	<0.0001
Adjuvant therapy	3.14	1.94–5.09	<0.0001	2.02	1.46–2.81	<0.0001
MSI	1.03	0.62–1.70	0.91	1.09	0.78–1.53	0.62
FGFR2 mutation	1.66	0.85–3.25	0.14	1.37	0.83–2.29	0.22
KRAS mutation	0.40	0.17–0.93	0.033	1.03	0.69–1.55	0.87
CTNNB1 mutation	0.58	0.28–1.22	0.15	0.70	0.44–1.11	0.13
PIK3CA mutation	0.74	0.40–1.38	0.34	0.71	0.47–1.08	0.11

Multivariate Analyses						
	Disease Free Survival*			Overall Survival**		
	HR Ratio	95% CI	P	HR Ratio	95% CI	P
FGFR2	1.83	0.90–3.73	0.097	1.34	0.79–2.27	0.28
KRAS	0.43	0.18–0.99	0.048	1.05	0.70–1.58	0.82
FGFR2^a^	1.64	0.80–3.36	0.18	1.37	0.80–2.33	0.25
KRAS^b^	0.45	0.19–1.06	0.067	1.08	0.71–1.63	0.73

*For DFS, the multivariate model included Stage 1C, II, III/IV, grade 2 and 3.

**For OS, the multivariate model included age, FIGO stage 1C, II, III/IV, grade 2 and grade 3.

a FGFR2 adjusted for KRAS in addition to covariates above.

b KRAS adjusted for FGFR2 in addition to covariates above.

The presence of *KRAS* mutation was associated with longer disease free survival (DFS) (HR = 0.40 95% CI 0.17–0.93; p = 0.03) whereas the mutation status of other genes was not significantly associated with DFS. As expected, DFS was associated with higher stage (III/IV) (p<0.0001), FIGO tumor grade 2 (p = 0.0019) and 3 (p<0.0001) and adjuvant therapy (p<0.0001) in univariate analysis. Multivariate analysis showed that the presence of a *KRAS* mutation remained significantly associated with longer DFS (HR = 0.43 95% CI 0.18–0.99; p = 0.048) (Table 2). When *FGFR2* mutation status was incorporated into a multivariate analysis it showed a trend towards being associated with shorter DFS (HR = 1.83 95% CI 0.90–3.73; p = 0.097) although this finding was of marginal statistical significance (Table 2). When both genes were included in a multivariate model neither reached significance (Table 2). *CTNNB1* and *PIK3CA* mutations had no effect on the multivariate model (data not shown). We did not include adjuvant therapy in the multivariate model as analysis indicated it was not independent of stage and grade.

### Mutations in early-stage disease and association with patient outcome

We then tested whether mutation status of any gene was associated with outcome in patients with early stage disease, defined as all stage I and II tumors. Univariate analysis revealed shorter OS is associated with age (p = 0.004), stage II (p = 0.007) and high tumor grade (FIGO grade 3) (p<0.0001) (Table 3). Both *FGFR2* mutation positivity and grade 2 differentiation showed a trend towards shorter OS (HR = 1.74; 95% CI 0.97–3.12; p = 0.065 and HR = 1.52; 95% CI 0.98–2.33; p = 0.059, respectively). When *FGFR2* mutation was analyzed taking into consideration the effects of known prognostic factors variables, it became more significantly associated with OS (HR = 2.00 95% CI 1.09–3.65; p = 0.025) (Table 3).

**Table 3:** Hazard ratio (HR) and 95% confidence interval (CI) for cohort of 386 Stage I/II cases.

Univariate Analyses						
	Disease Free Survival			Overall Survival		
	HR Ratio	95% CI	P	HR Ratio	95% CI	P
Age >60	1.42	0.69–2.92	0.35	1.92	1.23–3.00	0.004
Race (Black)	1.27	0.49–3.30	0.62	1.35	0.79–2.30	0.27
FIGO stage IA/1B	REF			REF		
FIGO stage IC	2.65	1.20–5.83	0.016	1.40	0.87–2.27	0.17
FIGO stage II	3.28	1.35–7.96	0.009	2.13	1.23–3.69	0.007
FIGO stage III/IV	1.56	0.70–3.50	0.27	1.52	0.98–2.33	0.059
FIGO Grade 2	4.49	1.92–10.50	0.0005	3.00	1.75–5.15	<0.0001
FIGO Grade 3	2.07	1.01–4.28	0.049	1.47	0.95–2.29	0.087
Adjuvant therapy	1.17	0.58–2.38	0.66	1.17	0.78–1.76	0.44
MSI	2.72	1.18–6.28	0.019	1.74	0.97–3.12	0.065
FGFR2 mutation	0.26	0.06–1.11	0.069	1.39	0.89–2.17	0.15
KRAS mutation	0.92	0.38–2.23	0.85	0.82	0.48–1.38	0.45
CTNNB1 mutation	0.69	0.28–1.66	0.40	0.77	0.47–1.24	0.27
PIK3CA mutation	1.42	0.69–2.92	0.35	1.92	1.23–3.00	0.004

Multivariate Analyses						
	Disease Free Survival*			Overall Survival**		
	HR Ratio	95% CI	P	HR Ratio	95% CI	P
FGFR2	3.24	1.35–7.77	0.008	2.00	1.09–3.65	0.025
KRAS	0.23	0.05–0.97	0.045	1.29	0.81–2.03	0.28
FGFR2^a^	3.03	1.26–7.27	0.013	2.05	1.12–3.75	0.021
KRAS^b^	0.24	0.06–1.02	0.053	1.31	0.83–2.07	0.25

*For DFS, the multivariate model included Stage 1C, II, Grade 2 and 3.

**For OS, the multivariate model included age, FIGO Stage 1C, II, Grade 2 and Grade 3.

a FGFR2 adjusted for KRAS in addition to covariates above.

b KRAS adjusted for FGFR2 in addition to covariates above.

Univariate analysis revealed only high grade (p = 0.0005); stage II (p = 0.009); adjuvant therapy (p = 0.049) and the presence of an *FGFR2* mutation (p = 0.019) were significantly associated with shorter disease free survival (DFS) (Table 3). *KRAS* mutation showed a trend towards associating with longer DFS (HR = 0.26 95% CI 0.06–1.11 p = 0.067) whereas *CTNNB1* and *PIK3CA* mutations were not associated with DFS. When each gene was analyzed alone in multivariate analysis of early stage cancers, *FGFR2* mutation status remained a significant factor associated with reduced DFS (HR = 3.24; 95% CI 1.35–7.77; p = 0.008) (Table 3) and *KRAS* was significantly associated with longer DFS (HR = 0.23 CI 0.05–0.97 p = 0.045). When both genes were included in the model, FGFR2 remained significant (HR = 3.03 CI 1.26–7.27 p = 0.013). Kaplan-Meier survival plots showing the relationship between *FGFR2* mutation and DFS and OS in early stage cancers are presented in Figure S1.

## Discussion

Here we show the patterns of mutations in four endometrial oncogenes in the largest cohort of endometrioid endometrial tumors reported to date (n = 466). Given the large number of tumors in this single institution Washington University School of Medicine cohort, novel insights have been revealed which have not been evident with smaller subsets of tumors or in some cases where disparate evidence had been reported in smaller panels of tumors [27], [28], [29], [30].

One finding that may have implications for understanding the biology underlying endometrial cancer is the hereto-unrecognized mutual exclusivity of *CTNNB1* and *KRAS* mutations in this cohort. Although 5 tumors were identified with mutations in both genes the vast majority of tumors only carried mutations in either *KRAS* or *CTNNB1* (p = 0.0002). This finding was not a reflection of an association with MSI positive and negative tumors because when we looked in only the MSS tumors, the association was even more significant. Only 1% *CTNNB1* mutation positive tumors carried a *KRAS* mutation whereas 19% of the *CTNNB1* wildtype tumors carried a *KRAS* mutation (p = 0.00004, two-tailed Fisher's exact test). In most other cancers, mutual exclusivity of gene activation is observed between two proteins that map to the same signaling pathway, which makes intuitive sense, as activation of the same pathway at two different nodes is redundant. Although *KRAS* and CTNNB1 have very distinct roles in the MAPK pathway and the Wnt/TCF signaling pathway respectively, recent data suggests novel points of pathway crosstalk in some cell types [31]. Additional work is needed to identify the mechanistic basis and biological significance of the mutual exclusivity of KRAS and CTNNB1 mutations in endometrial cancer. We hypothesize the presence of unappreciated crosstalk or a shared effector molecule between the two pathways in endometrial cells. Alternatively, the caveat exists that these two pathways do not demonstrate redundancy at the level of a shared effector molecule but perhaps merely demonstrate biological redundancy with regard to the functional effect activation of either pathway has on the tumorigenic phenotype. e.g. uncontrolled cellular proliferation.

In contrast to a previous study, our data suggest that mutations in exon 20 of *PIK3CA* are not associated with poor prognosis [29]. Since finalizing these analyses, it has been reported that mutations in exons 1–7 of PIK3CA are prevalent in endometrial cancer, and comprise 50% of all mutations identified [32]. Restricting mutation analysis to exons 9 and 20 is a limitation of the current study, and it is possible that thorough mutational analyses may yet reveal associations with clinicopathologic variables.

In this single institution series of endometrioid endometrial cancers, the overall *FGFR2* mutation rate was 10% (48/466). The 10% mutation rate for this large, unselected series is consistent with the mutation rate reported by Dutt et al. (9/86, 10%) [33] and Cheung et al. (24/243, 10%) [15]. In our initial report of *FGFR2* mutations in endometrial cancers we oversampled for cases that had recurred and tumors with microsatellite instability [18], which may explain in part the higher rate of mutations in that selected population, given the association of *FGFR2* mutation with both defective DNA repair and recurrence in the current unselected cohort.

A number of clinical and pathologic prognostic factors have been evaluated in the search for markers to more accurately predict risk of recurrence or death for patients with endometrial carcinoma. Past studies have suggested tumor markers p53, p16, estrogen receptor, progesterone receptor and HER2/neu may have clinical utility in endometrial cancer for predicting lymph node metastasis, prognosis and in directing treatment [34]; however, no molecular markers are routinely used clinically. Tumor aneuploidy has also been assessed and may be of some prognostic benefit for low grade cancers [35], however given its requirement for fresh tissue, it is not always clinically practical. An ongoing prospective multicenter study called Molecular Markers in Treatment in Endometrial Cancer (MoMaTEC) is currently accruing patients in Europe to investigate the predictive value of p53, p16, estrogen receptor, progesterone receptor and HER2/neu markers.

In this study we have identified that *FGFR2* and *KRAS* have prognostic significance within the cohort of endometrioid endometrial cancers. Our data suggest that *FGFR2* mutations occur more often in the well and moderately differentiated endometrioid tumors (G1, G2) compared to undifferentiated tumors and possibly identify the “bad actors” in an otherwise better prognosis histological subgroup. Recent data in an independent cohort of endometrial tumors reported a similar frequency of mutations across G1–G3 tumors [15]. This disparity could be explained by the fact that in that cohort, the pathogenicity of the identified mutations is uncertain as many were novel and their somatic status was not confirmed. A poorly differentiated histology was one of the strongest predictors of recurrence and/or progression in both the overall cohort and in all early stage cancers in both univariate and multivariate analyses, consistent with previous reports [2], [3], [5], [36]. Notably, the association of *FGFR2* with shorter DFS is more significant in the multivariate analyses where the association of high grade with poor prognosis is accounted for, compared to univariate analysis. These findings strongly suggest that the observed effect of FGFR2 is not simply due to the confounding effects of other known prognostic factors, and underscore the likely functional significance of this gene in determining survival.

A novel finding of this present study is that *KRAS* mutation is associated with longer DFS in the total cohort in both univariate and multivariate analysis. In the subset of early stage cases, *KRAS* mutation was significantly associated with longer DFS in multivariate analysis after adjusting for grade and stage. We can speculate that the pattern of mutual exclusivity of *FGFR2* and *KRAS* suggests that the role of these two genes in endometrial cancer initiation is likely to be through activation of the MAPK signaling pathway. The fact that they have different and indeed opposing effects on disease free survival leads us to further speculate that activation of “non-MAPK” pathways downstream of *FGFR2* is driving the association of this gene with poor prognosis.

**Figure 3:**
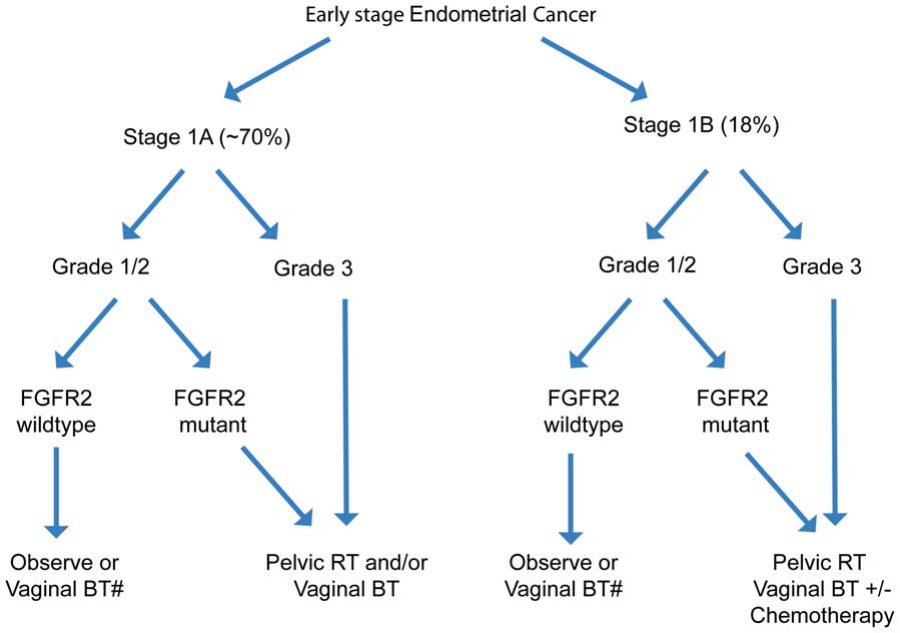
Potential utility of *FGFR2* mutation status as an adverse prognostic factor to affect clinical decision-making. The decision tree is adapted from 2011 National Comprehensive Cancer Network guidelines using FIGO 2009 staging. BT = brachytherapy; RT = radiation therapy.

Our finding that *FGFR2* mutation is an independent prognostic marker in patients with early stage endometrioid endometrial cancer suggests that *FGFR2* mutation testing could ultimately prove useful in the management of endometrial cancer. Current National Comprehensive Cancer Network (NCCN) guidelines for endometrioid endometrial cancer confined to the uterus recommends more aggressive adjuvant therapy as tumor grade and tumor stage increases, and also where multiple adverse prognostic indicators are present, including lymphovascular space involvement. We envisage that the mutation status of FGFR2 could be used to inform clinical decision making in a similar way to a poorly differentiated histology. Specifically, the presence of an FGFR2 mutation and absence of a KRAS mutation would stratify a patient as having high-risk disease, resulting in a recommendation for more aggressive therapy (See Figure 3).

Replication of this finding in an independent patient cohort is an important step in validating the potential clinical utility of FGFR2 as a prognostic marker. The key limitations to our current finding are 1) that the patient samples are from a single institution, 2) the frequency of recurrence in early stage endometrioid cases is relatively low in this unselected cohort and 3) we had low number of late stage G1 and G2 tumors in this cohort which may have contributed to lack of statistical significance for FGFR2 in the entire cohort. We are currently sequencing the four exons of FGFR2 containing almost all reported mutations in endometrial cancer samples collected as part of the multi-institutional GOG-210 clinical trial “Molecular Staging of Endometrial Cancer”. This cohort also allows the assessment of FGFR2 mutations on endometrial cancer specific survival as well as overall survival, given the extensive clinical annotation of these samples.

Preclinical data suggests that *FGFR2* mutation testing may identify patients whose tumors will be sensitive to FGFR inhibition [11], [37]. A large number of FGFR inhibitors are in development, preclinical studies, and clinical trials [38]. Currently, several multi-target kinase inhibitors with activity against multiple kinases including FGFRs are being evaluated in endometrial patients with advance stage or recurrent endometrial cancer (Brivinib, NCT00888173; E7080, NCT01111461, Dovitinib, NCT01379534) and additional trials with more specific FGFR inhibitors are planned. The validation of *FGFR2* mutations as an independent prognostic marker in early stage tumors and the eventual identification of an FGFR inhibitor with clinical activity in patients with metastatic endometrial cancer, holds the promise of utilizing anti-FGFR therapies in an adjuvant setting to reduce the risk of recurrence in patients diagnosed with *FGFR2* mutation positive endometrial cancer.

In conclusion, our mutation analysis of four oncogenes frequently mutated in the endometrioid histology of endometrial cancer revealed that mutated *FGFR2* was associated with shorter disease free progression and this was significant in patients diagnosed with early stage disease. This finding has clinical significance in that *FGFR2* mutation status could function as a starting point in developing a molecular prognostic risk assessment score that could be used to identify patients that may benefit from more aggressive adjuvant radiation and/or chemotherapy following an initial hysterectomy. In the longer term, anti-FGFR agents could be tested in patients with *FGFR2* mutation positive tumors to evaluate whether these agents reduce the frequency of recurrence in the adjuvant setting, in addition to the metastatic setting where they are currently being evaluated. As *KRAS* mutations were associated with reduced recurrence risk in this cohort, our data would suggest that MEK inhibition may not be effective in an adjuvant setting to prevent recurrence.

## Supporting Information

Figure S1 **Kaplan Meier curves for recurrence/progression free survival (A) and overall survival (B) by** ***FGFR2*** **mutation status in patients with early stage endometrial cancer.**
(TIF)

Table S1 **Clinicopathological features of endometrial tumors with** ***FGFR2*** **mutations.** **^a^**Numbering relative to NM_022970.2 **^b^**Numbering relative to NP_075259.2 **^c^**These mutations have been reported previously (8).
(DOC)

Table S2 ***KRAS*** **Mutations in Endometrial Tumors.**
(DOC)

Table S3 ***PIK3CA*** **Mutations in Endometrial Tumors.** ^#^These mutations are novel and do not appear in Cosmic (May 2011).
(DOC)

Table S4 ***CTNNB1*** **Mutations in Endometrial Tumors.**
(DOC)

Table S5 **Frequency of MSI and mutations, according to FIGO stage.**
(DOC)

Table S6 **Frequency of MSI and mutations, according to tumor grade.**
(DOC)
