# Correction: *FGFR2* Point Mutations in 466 Endometrioid Endometrial Tumors: Relationship with MSI, *KRAS*, *PIK3CA*, *CTNNB1* Mutations and Clinicopathological Features

**DOI:** 10.1371/annotation/0bfaecca-0f87-43fe-97cc-f2ae3ddeb6d5

**Published:** 2012-10-12

**Authors:** Sara A. Byron, Michael Gartside, Matthew A. Powell, Candice L. Wellens, Feng Gao, David G. Mutch, Paul J. Goodfellow, Pamela M. Pollock

There is an error in Table 3. A correct version of Table 3 can be seen here: 

**Figure:**
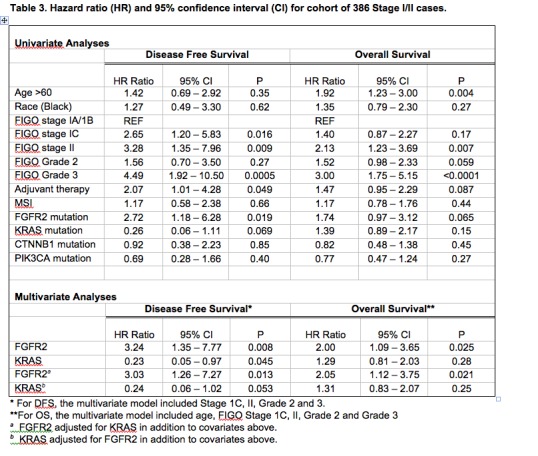


[^] 

