# Supplementary figures and images for: FGFR2 Point Mutations in 466 Endometrioid Endometrial Tumors: Relationship with MSI, KRAS, PIK3CA, CTNNB1 Mutations and Clinicopathological Features
Source: PLoS One. 2012 Feb 23;7(2):e30801. doi: 10.1371/journal.pone.0030801 (PMC3285611; doi:10.1371/journal.pone.0030801)

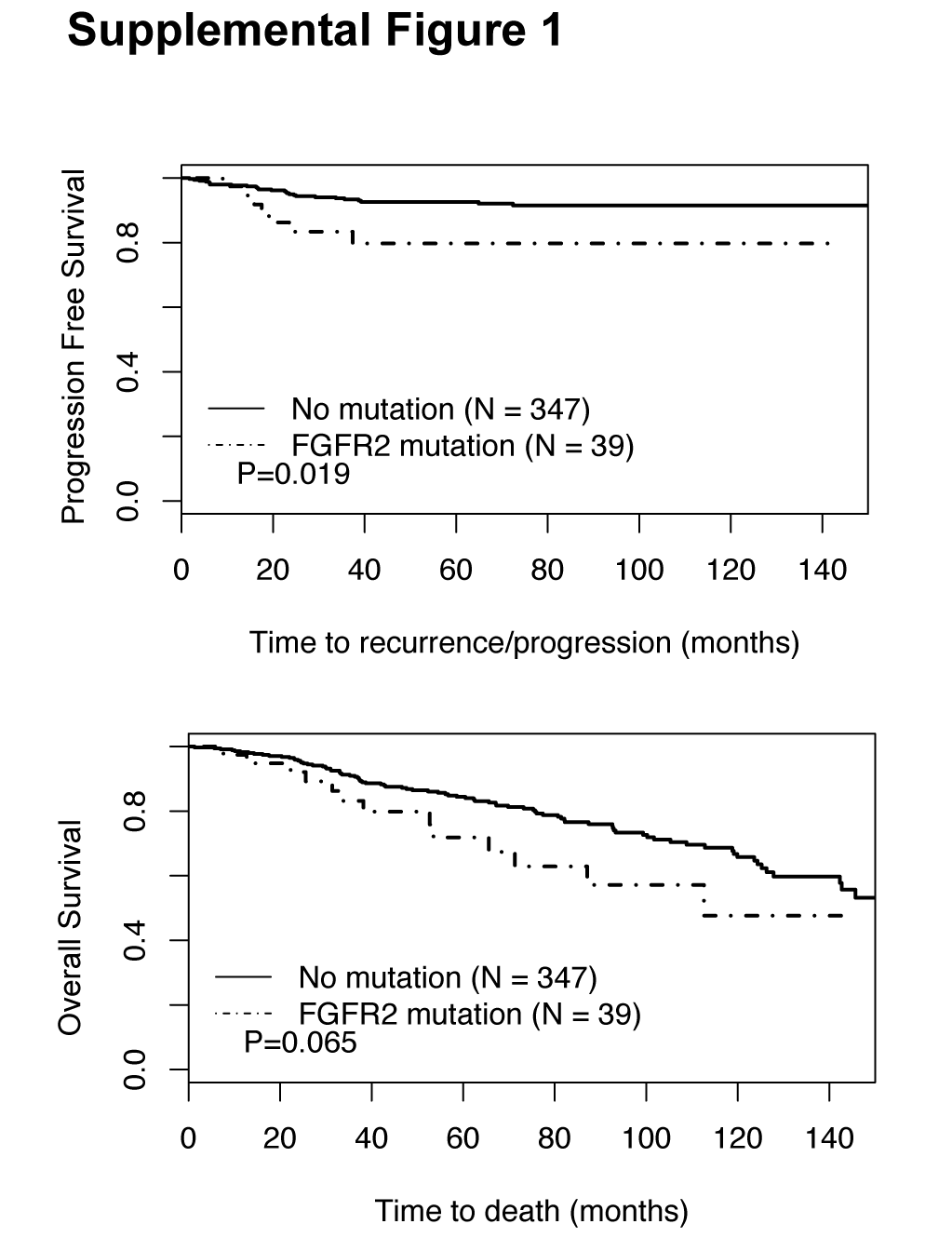

Supplement: Figure S1 — Kaplan Meier curves for recurrence/progression free survival (A) and overall survival (B) by FGFR2 mutation status in patients with early stage endometrial cancer. (TIF) [file pone.0030801.s001.tif]
