# Supplementary material for: FGFR2 Point Mutations in 466 Endometrioid Endometrial Tumors: Relationship with MSI, KRAS, PIK3CA, CTNNB1 Mutations and Clinicopathological Features
Source: PLoS One. 2012 Feb 23;7(2):e30801. doi: 10.1371/journal.pone.0030801 (PMC3285611; doi:10.1371/journal.pone.0030801)
Supplement: Table S1 — Clinicopathological features of endometrial tumors with FGFR2 mutations. aNumbering relative to NM_022970.2 bNumbering relative to NP_075259.2 cThese mutations have been reported previously (8). (DOC) [file pone.0030801.s002.doc]

**Table S1. Clinicopathological features of endometrial tumors with *FGFR2* mutations.**

| **Case ID** | **Stage** | **Grade** | **Recur** | ***FGFR2*b DNA sequence***^a^* | **Codon Change** |
| --- | --- | --- | --- | --- | --- |
|  |  |  |  |  |  |
| 1133 | IA | 1 | N | c.755C>G | p.S252W |
| 1141 | IB | 2 | N | c.755C>G | p.S252W |
| 1195 | IA | 1 | N | c.755C>G | p.S252W |
| 1410 | IIIC | 2 | N | c.755C>G | p.S252W |
| 1431 | IIA | 1 | N | c.755C>G | p.S252W |
| 1536 | IA | 1 | N | c.755C>G | p.S252W |
| 1604 | IA | 1 | N | c.755C>G | p.S252W |
| 1806 | IB | 1 | N | c.755C>G | p.S252W |
| 1829 | IC | 1 | N | c.755C>G | p.S252W |
| 1958 | IC | 1 | Y | c.755C>G | p.S252W |
| 1987 | IB | 1 | N | c.755C>G | p.S252W |
| 1359**^c^** | IB | 2 | Y | c.755C>G | p.S252W |
| 1574**^c^** | IC | 2 | Y | c.755C>G | p.S252W |
| 1484**^c^** | IIIC | 3 | Y | c.755C>G | p.S252W |
| 1316**^c^** | IIIC | 1 | Y | c.755C>G | p.S252W |
| 1792**^c^** | IIIC | 1 | N | c.755C>G | p.S252W |
| 1482**^c^** | IVA | 2 | N | c.755C>G | p.S252W |
| 1130 | IC | 1 | N | c.758C>G | p.P253R |
| 1590 | IB | 2 | N | c.758C>G | p.P253R |
| 1684**^c^** | IB | 1 | N | c.1118C>G | p.S373C |
| 1363 | IB | 2 | N | c.1127A>G | p.Y376C |
| 1655**^c^** | IIIC | 2 | Y | c.1127A>G | p.Y376C |
| 1361**^c^** | IB | 1 | Y | c.1175T>G | p.M392R |
| 2033 | IB | 1 | N | c.1187_1188delinsAT | p.V396D |
| 1524 | IC | 2 | N | c.1192C>A | p.L398M |
| 1744**^c^** | IIIC | 2 | N | c.1642A>G | p.I548V |
| 1220 | IB | 1 | N | c.1650T>A | p.N550K |
| 1231 | IB | 1 | N | c.1650T>A | p.N550K |
| 1249 | IB | 1 | N | c.1650T>A | p.N550K |
| 1347 | IB | 1 | N | c.1650T>A | p.N550K |
| 1464 | IIA | 3 | N | c.1650T>A | p.N550K |
| 1631 | IIB | 1 | N | c.1650T>A | p.N550K |
| 1714 | IA | 1 | N | c.1650T>A | p.N550K |
| 1877 | IIA | 1 | N | c.1650T>A | p.N550K |
| 1946 | IIB | 1 | N | c.1650T>G | p.N550K |
| 1267**^c^** | IIA | 2 | Y | c.1650T>A | p.N550K |
| 1391**^c^** | IIIC | 2 | N | c.1650T>A | p.N550K |
| 1528**^c^** | IVA | 2 | N | c.1650T>A | p.N550K |
| 2066 | IB | 2 | N | c.1648A>C | p.N550H |
| 1550 | IB | 1 | N | c.1978A>G | p.K660E |
| 1587 | IC | 1 | N | c.1978A>G | p.K660E |
| 2024 | IB | 2 | N | c.1978A>G | p.K660E |
| 1717**^c^** | IC | 2 | N | c.1978A>G | p.K660E |
| 1164 | IC | 2 | N | c.1147T>C | p.C383R |
| 1729 | IA | 1 | N | c.1147T>C | p.C383R |
| 1094**^c^** | IB | 1 | Y | c.1147T>C | p.C383R |
| 1492**^c^** | IC | 1 | Y | c.[755C>GC755G(+)1127A>G]A1127G | p.[S252W(+) Y376C] |
| 1272**^c^** | IA | 1 | N | Intron10 A>C+2 |  |
