# Supplementary material for: FGFR2 Point Mutations in 466 Endometrioid Endometrial Tumors: Relationship with MSI, KRAS, PIK3CA, CTNNB1 Mutations and Clinicopathological Features
Source: PLoS One. 2012 Feb 23;7(2):e30801. doi: 10.1371/journal.pone.0030801 (PMC3285611; doi:10.1371/journal.pone.0030801)
Supplement: Table S2 — KRAS Mutations in Endometrial Tumors. (DOC) [file pone.0030801.s003.doc]

**Table S2.** ***KRAS* Mutations in Endometrial Tumors.**

| Exon | DNA sequence | Codon Change | # of Tumors (N=87/464). |
| --- | --- | --- | --- |
|  |  |  |  |
| 2 | c.34G>T | G12C | 9 |
| 2 | c.34G>A | G12S | 2 |
| 2 | c.34G>C | G12R | 1 |
| 2 | c.35G>A | G12D | 29 |
| 2 | c.35G>T | G12V | 25 |
| 2 | c.35G>C | G12A | 8 |
| 2 | c.38G>A | G13D | 13 |
|  |  |  |  |
