# Supplementary material for: FGFR2 Point Mutations in 466 Endometrioid Endometrial Tumors: Relationship with MSI, KRAS, PIK3CA, CTNNB1 Mutations and Clinicopathological Features
Source: PLoS One. 2012 Feb 23;7(2):e30801. doi: 10.1371/journal.pone.0030801 (PMC3285611; doi:10.1371/journal.pone.0030801)
Supplement: Table S3 — PIK3CA Mutations in Endometrial Tumors. #These mutations are novel and do not appear in Cosmic (May 2011). (DOC) [file pone.0030801.s004.doc]

**Table S3.** ***PIK3CA* Mutations in Endometrial Tumors.**

| **Exon** | DNA sequence | *PIK3CA*  codon change | # of Tumors (n=104/464) |
| --- | --- | --- | --- |
|  |  |  |  |
| 9 | c.1625A>G | E542G | 1 |
| 9 | c.1624G>A | E542K | 11 |
| 9 | c.1624G>C | E542Q | 1 |
| 9 | c.1625A>T | E542V | 1 |
| 9 | c.1634A>C | E545A | 4 |
| 9 | c.1635G>C | E545D | 1 |
| 9 | c.1634A>G | E545G | 3 |
| 9 | c.1633G>A | E545K | 13 |
| 9 | c.1636C>A | Q546K | 2 |
| 9 | c.1637A>T | Q546L | 1 |
| 9 | c.1637A>G | Q546R | 1 |
| 20 | c.2946G>T | E982D | 1 |
| 20 | c.3016C>T | L1006F^#^ | 2 |
| 20 | c.3042A>C | Q1014H^#^ | 1 |
| 20 | c.3062A>G | Y1021C | 8 |
| 20 | c.3073A>T | T1025S | 2 |
| 20 | c.3073A>G | T1025A | 1 |
| 20 | c.3129G>T | M1043I | 1 |
| 20 | c.3127A>G | M1043V | 1 |
| 20 | c.3140A>T | H1047L | 5 |
| 20 | c.3140A>G | H1047R | 23 |
| 20 | c.3139C>T | H1047Y | 5 |
| 20 | c.3155C>A | T1052K | 1 |
| 20 | c.3172A>C | I1058L | 1 |
| 20 | c.3193C>A | H1065Y | 2 |
| 9, 20 | c.1624G>A; c.3132T>G | E542K,N1044K | 1 |
| 9, 20 | c.1624G>A; c.3139C>T | E542K,H1047Y | 1 |
| 9, 20 | c.1635G>C; c.3012G>T | E545D,M1004I | 1 |
| 20 | c.3073A>G; c.3085G>T | T1025A, D1029Y^#^ | 1 |
|  |  |  |  |
