# Supplementary material for: FGFR2 Point Mutations in 466 Endometrioid Endometrial Tumors: Relationship with MSI, KRAS, PIK3CA, CTNNB1 Mutations and Clinicopathological Features
Source: PLoS One. 2012 Feb 23;7(2):e30801. doi: 10.1371/journal.pone.0030801 (PMC3285611; doi:10.1371/journal.pone.0030801)
Supplement: Table S4 — CTNNB1 Mutations in Endometrial Tumors. (DOC) [file pone.0030801.s005.doc]

**Table S4. *CTNNB1* Mutations in Endometrial Tumors.**

| Exon | DNA sequence | Codon Change | # of Tumors (N=88/454). |
| --- | --- | --- | --- |
|  |  |  |  |
| 3 | c.88-99(del12) | p.Tyr30_S33del | 1 |
| 3 | c.95A>G | D32G | 1 |
| 3 | c.94G>A | D32N | 9 |
| 3 | c.94G>T | D32Y | 11 |
| 3 | c.97T>C | S33A | 2 |
| 3 | c.98C>G | S33C | 10 |
| 3 | c.98C>T | S33F | 3 |
| 3 | c.97T>C | S33P | 1 |
| 3 | c.98C>A | S33Y | 4 |
| 3 | c.101G>A | G34E | 4 |
| 3 | c.101G>T | G34V | 1 |
| 3 | c.109T>G | S37A | 1 |
| 3 | c.110C>G | S37C | 6 |
| 3 | c.110C>T | S37F | 15 |
| 3 | c.109T>C | S37P | 1 |
| 3 | c.121A>G | T41A | 4 |
| 3 | c.122C>T | T41I | 6 |
| 3 | c.122C>A | T41N | 2 |
| 3 | c.134C>G | S45C | 1 |
| 3 | c.134C>T | S45F | 4 |
| 3 | c.157G>A | E54K | 1 |
|  |  |  |  |
