# Supplementary material for: FGFR2 Point Mutations in 466 Endometrioid Endometrial Tumors: Relationship with MSI, KRAS, PIK3CA, CTNNB1 Mutations and Clinicopathological Features
Source: PLoS One. 2012 Feb 23;7(2):e30801. doi: 10.1371/journal.pone.0030801 (PMC3285611; doi:10.1371/journal.pone.0030801)
Supplement: Table S5 — Frequency of MSI and mutations, according to FIGO stage. (DOC) [file pone.0030801.s006.doc]

**Table S5. Frequency of MSI and mutations, according to FIGO stage.**

| FIGO Stage | MSI | *FGFR2* | *KRAS* | *CTNNB1* | *PIK3CA* |
| --- | --- | --- | --- | --- | --- |
|  |  |  |  |  |  |
|  |  |  |  |  |  |
| IA | 19/85 (22%) | 7/85 (8%) | 19/85 (22%) | 20/84 (24%) | 19/84 (23%) |
| IB | 70/192 (36%) | 17/192 (9%) | 31/191 (16%) | 34/183 (19%) | 46/191 (24%) |
| IC | 30/71 (41%) | 9/71 (13%) | 18/70 (24%) | 15/71 (21%) | 19/71 (27%) |
| IIA | 8/18 (44%) | 4/18 (22%) | 4/18 (22%) | 2/18 (11%) | 5/18 (28%) |
| IIB | 2/20 (10%) | 2/20 (10%) | 1/20 (5%) | 7/20 (35%) | 4/20 (20%) |
| III | 24/62 (39%) | 7/62 (11%) | 13/62 (21%) | 9/61 (15%) | 9/62 (15%) |
| IV | 5/18 (28%) | 2/18 (11%) | 1/18 (6%) | 1/17 (6%) | 2/18 (12%) |
|  |  |  |  |  |  |
