# Supplementary material for: FGFR2 Point Mutations in 466 Endometrioid Endometrial Tumors: Relationship with MSI, KRAS, PIK3CA, CTNNB1 Mutations and Clinicopathological Features
Source: PLoS One. 2012 Feb 23;7(2):e30801. doi: 10.1371/journal.pone.0030801 (PMC3285611; doi:10.1371/journal.pone.0030801)
Supplement: Table S6 — Frequency of MSI and mutations, according to tumor grade. (DOC) [file pone.0030801.s007.doc]

**Table S6. Frequency of MSI and mutations, according to tumor grade**

| Grade | MSI | *FGFR2* | *KRAS* | *CTNNB1* | *PIK3CA* |
| --- | --- | --- | --- | --- | --- |
|  |  |  |  |  |  |
| 1 | 67/249 (27%) | 29/249 (12%) | 45/248 (18%) | 59/243 (24%) | 55/248 (22%) |
| 2 | 74/152 (49%) | 17/152 (11%) | 33/151 (22%) | 25/149 (17%) | 38/151 (25%) |
| 3 | 17/65 (26%) | 2/65 (3%) | 9/65 (14%) | 4/62 (6%) | 11/65 (17%) |
|  |  |  |  |  |  |
